# Case identification of non-traumatic brain injury in youth using linked population data

**DOI:** 10.1186/s12883-024-03575-6

**Published:** 2024-03-02

**Authors:** Rebecca F Slykerman, Betony E Clasby, Jimmy Chong, Kathryn Edward, Barry J Milne, Helen Temperton, Hiran Thabrew, Nicholas Bowden

**Affiliations:** 1 Department of Psychological Medicine, Te Ara Hāro, University of Auckland, Building 507, 22-30 Park Avenue, Auckland, Grafton 1023 New Zealand; 2 Department of Women’s and Children’s Health, University of Otago, Dunedin, New Zealand; 3 Paediatric Rehabilitation Service, Te Whatu Ora, Te Toka Tumai, Auckland, New Zealand; 4 Centre of Methods and Policy Application in the Social Sciences, University of Auckland, Auckland, New Zealand

**Keywords:** Administrative data, Non-traumatic brain injury, Case identification, Integrated data infrastructure

## Abstract

**Background:**

Population-level administrative data provides a cost-effective means of monitoring health outcomes and service needs of clinical populations. This study aimed to present a method for case identification of non-traumatic brain injury in population-level data and to examine the association with sociodemographic factors.

**Methods:**

An estimated resident population of youth aged 0–24 years was constructed using population-level datasets within the New Zealand Integrated Data Infrastructure. A clinical consensus committee reviewed the International Classification of Diseases Ninth and Tenth Editions codes and Read codes for inclusion in a case definition. Cases were those with at least one non-traumatic brain injury code present in the five years up until 30 June 2018 in one of four databases in the Integrated Data Infrastructure. Rates of non-traumatic brain injury were examined, both including and excluding birth injury codes and across age, sex, ethnicity, and socioeconomic deprivation groups.

**Results:**

Of the 1 579 089 youth aged 0–24 years on 30 June 2018, 8154 (0.52%) were identified as having one of the brain injury codes in the five-years to 30 June 2018. Rates of non-traumatic brain injury were higher in males, children aged 0–4 years, Māori and Pacific young people, and youth living with high levels of social deprivation.

**Conclusion:**

This study presents a comprehensive method for case identification of non-traumatic brain injury using national population-level administrative data.

**Supplementary Information:**

The online version contains supplementary material available at 10.1186/s12883-024-03575-6.

## Background

Acquired brain injury (ABI) is the umbrella term for brain injuries that occur after birth and affect the physical integrity, metabolic activity, or functional ability of neurons in the brain [1]. ABI can be categorised according to the mechanism of injury. Traumatic brain injury (TBI) occurs when an alteration in brain function follows an external force exerted on the head as in a fall, road traffic accident, or sports injury. By contrast, non-traumatic acquired brain injury (NT-ABI) is usually caused by internal factors such as a lack of oxygen following asphyxiation or vascular rupture; exposure to toxins following poisoning or infections such as encephalitis or meningitis; or pressure-related tissue damage (e.g. due to brain tumours).

ABI during childhood can result in a range of subtle to profound consequences across the lifespan. Severe ABI at a younger age is associated with a raft of neurocognitive deficits in processing speed, attention, verbal and non-verbal problem-solving skills, memory and executive function [2, 3]. These deficits can affect a child at home, school and in the community [4]. Longer-term adverse outcomes include poorer educational achievement, lower employment status, and increased mental health problems [5]. Compared to those who sustain a TBI, both adults and children with NT-ABI achieve less functional and cognitive recovery than those with TBI following multidisciplinary neurorehabilitation [6–8]. This may partly be explained by the greater number of comorbid conditions in NT-ABI [8].

Longitudinal research to investigate later life sequelae and support needs of an NT-ABI in childhood incurs a significant participant burden and is expensive to conduct. There is limited data regarding outcomes for children and young people who collectively fall within the NT-ABI group. Observational studies conducted in specific types of NT-ABI such as those with encephalitis, brain tumours, or cardiac arrest suggest high rates of neuropsychological and functional impairment [9–11].

Population level administrative data provides a cost-effective method of examining health outcomes and monitoring trends among specific clinical groups such as those with NT-ABI. Standardized case definitions and a process for case identification using the definition is fundamental to the use of administrative data for these research purposes [12, 13]. Researchers have proposed case identification methodologies across the TBI severity spectrum and grappled with the challenges of data validation and agreement about specific classification codes, and these have primarily been focused on TBI rather than NT-ABI [14, 15].

Proposed case definitions for TBI have been published using the International Classification of Diseases Tenth Edition (ICD-10) codes and these have been investigated in hospital level administrative data in adults [16] and children [17]. Chen and Colantonio (2011) defined TBI using a set of ICD-10 codes by first completing a literature review of papers that used an ICD10 definition of brain injury or spinal cord injury. Codes that were consistently used across studies, had a strong correlation with brain injury, or fitted with the theoretical framework of brain injury were considered by a committee for inclusion in the final case definition [18]. The definition was then applied in an investigation of delayed discharge from hospital days in TBI patients alongside those with NT-ABI. In this paper ICD-10 codes for anoxia, brain tumours, encephalitis, metabolic encephalopathy, and vascular insults excluding stroke were included to capture NT-ABI. Further detail about the process for determining which NT-ABI codes to include was not described [19]. An investigation of service utilization in NT-ABI children and youth used ICD-10 codes from the definition of NT-ABI produced by the Commission for Accreditation of Rehabilitation Facilities (CARF). Healthcare service utilization was found to be high in the pediatric NT-ABI population [20]. To date, defining NT-ABI and a process for determining the inclusion of relevant codes that can be applied in population-level administrative data is scarce relative to the literature for TBI.

A large research project that developed a case identification method for neurodiversity and TBI (Clasby et al. forthcoming work) and led to a case identification method for common childhood mental health disorders using routinely collected population level data [21] presented an opportunity to build on this work and establish a method for case identification for NT-ABI. The objective of the current study was to develop a case identification method for NT-ABI in child and youth populations using linked administrative data through clinical consensus of diagnostic codes. A secondary objective was to explore an application of this method over a five-year period to determine how many individuals were identified with NT-ABI overall, and by sociodemographic subgroup. Estimating prevalence of NT-ABI was not an objective of this study.

## Methods

### Integrated data infrastructure

Routine health and demographic data were obtained from the Integrated Data Infrastructure (IDI), a collection of whole-population administrative data sources linked at the individual level and managed by the New Zealand Government agency Statistics New Zealand [22, 23]. To protect data privacy, IDI data are deidentified and accessed only through secure ‘Datalabs’: facilities restricted to approved users, with access to data through a secure connection to a dedicated server. Statistics New Zealand protocols require all results to be aggregated and reviewed for confidentiality prior to release from the Datalab environment.

### Population-level databases within the IDI

#### National minimum data set (NMDS)

The NMDS contains New Zealand hospital admission data from all publicly funded hospitals in New Zealand. This includes emergency department presentations and day patient admissions exceeding three hours. The NMDS contains International Classification of Diseases (ICD) codes identifying both primary and secondary diagnoses. The ICD is a system used to code diagnostic and procedural information associated with hospital admissions. ICD codes can be used for payment and funding allocation, population level disease monitoring, and research investigating trends in disease. The ICD-9 Clinical modification (ICD-9 CM) was used to classify mortality causes in the United States until it was replaced by the updated ICD-10. An Australian modification of the ICD-10 (ICD-10 AM) was first released in 1998 and used in this case identification study to identify codes relevant in the New Zealand context of the IDI.

The inclusion of secondary diagnoses in the NMDS allows for case identification in situations where the brain injury is not the primary reason for admission.

#### Socrates

Socrates is a Ministry of Health database that contains information about use of disability support services. For example when people with a disability are referred to or seen by a Needs Assessment Service Coordinator (NASC) to ascertain eligibility and need for services such as in-home help, this is recorded in Socrates. An individual’s record contains diagnosis information from referral to services made by General Practitioners, Paediatricians, and other medical specialists that is used in Socrates specific diagnosis codes. If a child has more than one diagnosis these are all captured in the Socrates coding from the referral.

#### Accident compensation corporation (ACC)

The Accident Compensation Corporation is a government agency that funds injury related needs arising as the result of an accident, and is available to all New Zealanders. Non-residents injured while in New Zealand are also eligible for ACC. The database contains information about claims made following injury. In New Zealand, children who sustain a TBI in an accident have their support needs funded through ACC. Children who acquire a non-traumatic brain injury for example after a stroke, infection of the brain, brain tumour or cardiac arrest are not typically covered by ACC and instead access support via multiple agencies including the Ministry of Health, the Ministry of Education, disability support services and community mental health services. Approved treatment-related injuries are covered by ACC and therefore some children who suffer a non-traumatic brain injury as the result of rare or unexpected complications to a medical procedure or intervention, or preventable delay in access necessary care can lodge claims with ACC. Data within this dataset includes ICD-9, ICD-10, and Read diagnosis codes that pertain to the injury that is being claimed for. Read codes are a standardized and simplified set of codes developed in the United Kingdom and first applied in the National Health Service in 1985. They are used to assign diagnoses and injury categories by a health practitioner and are not country-specific [24]. Read codes assist in consistency of terminology across systems capturing symptom, diagnostic, and procedural information. In New Zealand Read codes are used in systems capturing information about injury related claims for healthcare.

#### Programme for the integration of mental health data (PRIMHD)

The PRIMHD is a database containing information from providers of specialist mental health services. These services are provided by Non-Government Organisations (NGO) and by Government funded community mental health services located within each health district of New Zealand. PRIMHD includes ICD-10 and Diagnostic and Statistical Manual for Mental Disorders – Fourth Edition (DSM-4) primary and secondary diagnosis codes. The DSM-4 is the official manual of the American Psychiatric Association and a provides diagnostic criteria for classifying psychiatric disorders. Exploratory analysis showed no cases of NT-ABI were found in the PRIMHD dataset.

### Case identification

Firstly, preliminary lists of ICD-9 CM, ICD-10 AM, Socrates specific diagnosis codes, and Read codes were constructed. We included birth trauma codes in our case identification process. Different definitions of ABI treat inclusion of birth trauma differently, therefore we include these as a distinct sub-group so that these codes can be separated out if desired.

A clinical consensus committee was formed and comprised of five health professionals with clinical experience in acquired brain injury (two Pediatric Rehabilitation Medicine Specialists, two Child and Adolescent Psychiatrists, and a Pediatric Neuropsychologist). All members of the group had > 10 years of experience working with young people following acquired brain injury, were currently working in clinical practice in young people with brain injury, and had training in brain development, mechanisms of injury, and treatment/intervention. Utilising a clinical consensus approach has successfully been applied to identification of youth mental health problems [21]. Each member of the group systematically evaluated each code (including both ICD and Read codes) to determine if it indicated an acquired brain injury. The overall aim of the clinical consensus process was to establish a set of codes that if present would mean a brain injury was most likely to have occurred.

Codes were included if they clearly indicated damage to the brain had occurred (e.g. codes that included hypoxic injury, or indicated brain dysfunction e.g. encephalitis or encephalopathy), or if brain structures were identified as being affected (e.g. codes that specified the location of brain tumours). Codes for conditions where there was a possibility that an injury to the brain could occur as sequelae of the condition or event were not included if the code did not identify or define an injury to the brain at the time of the event (e.g. toxicity from poisoning that did not specify brain involvement in the code description). The rate of initial agreement between committee members was 98% for ICD-10AM codes (agreement on 218 of 223), 97% for ICD-9CM codes (168 of 174), and 89% for Read codes (agreement on 796 of 898). In the cases of initial discrepancy in agreement this was resolved by group discussion until a consensus opinion was reached. Specifically the process of reaching agreement involved: reviewing the criteria for inclusion, committee members providing their rationale for their initial decision, and review of the inclusion decision for similar codes. In all cases this process resulted in unanimous agreement between committee members. Inclusion decisions for codes were then checked for consistent application across the ICD-9 CM, ICD-10 AM, and Read codes. The full list of included codes is presented in supplementary tables S1-S4.

Using date of service use recorded in each dataset, a person was identified as an NT-ABI case if at least one of the NT-ABI codes was present in the five years up until 30 June 2018 in one of the four databases (NMDS, ACC, Socrates, PRIMHD).

### Sociodemographic measures

Sex was classified as male or female. Age was calculated in years and months as at 30 June 2018. Ethnicity was categorised as ‘European’, ‘Māori’, ‘Pacific’, ‘Asian’, ‘Middle Eastern, Latin American or African’ (MELAA), and ‘Other’ using a ‘total ethnicity’ approach that allows people to indicate all ethnic groups with which they identify. In New Zealand it is common for people to identify with more than one ethnic group [25]. The total ethnicity approach results in the capture of all ethnic groups a person identifies with and therefore individuals are not assigned a single mutually exclusive ethnicity category. The New Zealand Deprivation Index (NZDep) is a comprehensive measure of socioeconomic status that encompasses economic deprivation markers including income, home ownership, and housing information. Higher NZ Dep scores indicate greater levels of economic deprivation. Using the residential address database from the IDI at 30 June 2018, NZDep scores were merged at the meshblock (neighbourhood) level and collapsed to quintiles. A binary measure of geographical location (urban or rural) was used with rural dwelling defined as locations with populations < 1,000 people.

### Data analysis

Preparation of data was conducted using SAS Enterprise Guide version 8.3 and analysis was undertaken in Stata MP version 16.1 in the secure IDI environment. The New Zealand estimated resident population (ERP) of young people aged between 0 and 24 years in the 2017/2018 fiscal year was created using an established IDI method [26, 27]. This method aims to include all those who were alive and living in New Zealand as at 30 June 2018. The method constructs an ERP that is within 2% of the official ERP [27].

Population rates of NT-ABI are presented overall and by sociodemographic sub-group. Rates excluding birth trauma events, and rates of birth trauma NT-ABI are also presented separately. In addition, NT-ABI cases by data source of identification are presented.

## Results

### Overall rates of NT-ABI identified

A total of 1 579 089 youth aged 0–24 years at 30 June 2018 formed the cohort of children and adolescents resident in New Zealand in the 2017/2018 fiscal year. Of those, 8154 (0.52%) were identified as having one of the NT-ABI codes in the five year period prior to 30 June 2018 equivalent to 516 per 100 000. Table 1 shows the five year rates of non-traumatic acquired brain injury for the population and broken down by inclusion of birth trauma codes. Males had slightly higher rates of NT-ABI than females. The overall rate of NT-ABI was highest in children aged 0–4 years (1352 per 100 000) compared to 344 per 100 000 in 5–9 year olds, 236 per 100 000 in 10–14 year olds, 342 per 100 000 in those aged 15–19 years, and 366 per 100 000 in 20–24 year olds.

**Table 1:** Case identification of non-traumatic acquired brain injury by demographic characteristics

		Total NT-ABI cases		NT-ABI cases excl. birth trauma		Birth Trauma	
		N	%	N	%	N	%
**Total Population**	1,579,089	8154	0.52	6057	0.38	2265	0.14
**Sex**							
Male	813 123	4530	0.56	3345	0.41	1287	0.16
Female	765 966	3624	0.47	2712	0.35	978	0.13
**Age group**							
0–4 years	296 814	4014	1.35	1938	0.65	2241	0.76
5–9 years	323 043	1110	0.34	1089	0.34	24	0.01
10–14 years	311 826	735	0.24	735	0.24	n/a	
15–19 years	309 972	1059	0.34	1059	0.34	n/a	
20–24 years	337 434	1236	0.37	1236	0.37	n/a	
**Ethnicity**							
European	1 064 181	5454	0.51	4089	0.38	1467	0.14
Māori	408 150	2949	0.72	2214	0.54	789	0.19
Pacific	212 823	1425	0.67	1050	0.49	405	0.19
Asian	270 312	900	0.33	603	0.22	318	0.12
MELAA	32 259	153	0.47	111	0.34	48	0.15
**Deprivation Index**							
Lowest quintile	293 109	1152	0.39	906	0.31	270	0.09
Dep2	277 701	1218	0.44	930	0.33	312	0.11
Dep3	277 389	1314	0.47	969	0.35	372	0.13
Dep4	291 522	1617	0.55	1158	0.40	501	0.17
Highest quintile	355 167	2478	0.70	1797	0.51	726	0.20
**Resident location**							
Urban	1306 389	6807	0.52	5061	0.39	1890	0.14
Rural	262 071	1305	0.50	966	0.37	363	0.14

MELAA: Middle Eastern, Latin American, African

NT-ABI: non-traumatic acquired brain injury

Differences in rates of ABI by ethnicity were evident with higher relative rates in Māori (722 per 100 000) and Pacific (669 per 100 000) and lower rates in Asian (332 per 100 000)and MELAA (480 per 100 000) young people. There was a clear deprivation gradient and rates of NT-ABI were highest amongst those living in the most deprived areas (697 per 100 000). These economic and ethnic differences were evident in both rates of birth trauma and in non-birth trauma cases of NT-ABI.

### Source of cases identified

Table 2 shows the source of cases identified stratified by dataset and by sociodemographic sub-group. The NMDS contained 87.2% of the cases identified with non-traumatic brain injury. Excluding birth trauma codes, 82.9% of cases were identified in the NMDS while birth trauma cases came exclusively from the NMDS). The second highest number of NT-ABI cases were found in the ACC database at 15.3%. A small number of cases were identified in the Socrates dataset (1.7%).

**Table 2:** Dataset sources of case identification of NT-ABI

	Total NT-ABI cases identified						NT-ABI Cases Excluding Birth Trauma						Birth Trauma	
	NMDS		ACC		SOCRATES		NMDS		ACC		SOCRATES		NMDS	
	N	%	N	%	N	%	N	%	N	%	N	%	N	%
**Total Population**	7161	87.8	1248	15.3	135	1.7	5019	82.9	1248	20.6	135	2.2	2265	100.0
**Sex**														
Male	3963	87.5	711	15.7	84	1.9	2751	82.2	711	21.3	84	2.5	1287	100.0
Female	3198	88.2	537	14.8	51	1.4	2268	83.6	537	19.8	51	1.9	978	100.0
**Age group**														
0–4 years	3771	93.9	360	9.0	24	0.6	1650	85.1	360	18.6	24	1.2	2241	100.0
5–9 years	849	76.5	318	28.6	36	3.2	828	76.0	318	29.2	36	3.3	24	100.0
10–14 years	576	78.4	168	22.9	30	4.1	576	78.4	168	22.9	30	4.1	n/a	
15–19 years	894	84.4	192	18.1	24	2.3	894	84.4	192	18.1	24	2.3	n/a	
20–24 years	1074	86.9	207	16.7	21	1.7	1074	86.9	207	16.7	21	1.7	n/a	
**Ethnicity**														
European	4713	86.4	906	16.6	84	1.5	3324	81.3	906	22.2	84	2.1	1467	100.0
Māori	2616	88.7	441	15.0	51	1.7	1872	84.6	441	19.9	51	2.3	789	100.0
Pacific	1287	90.3	186	13.1	30	2.1	912	86.9	186	17.7	30	2.9	405	100.0
Asian	807	89.7	111	12.3	18	2.0	504	83.6	111	18.4	18	3.0	318	100.0
MELAA	132	86.3	30	19.6	.S		84	75.7	30	27.0	.S		48	100.0
**Deprivation Index**														
Lowest quintile	984	85.4	201	17.4	18	1.6	726	80.1	201	22.2	18	2.0	270	100.0
Dep2	1053	86.5	210	17.2	18	1.5	756	81.3	210	22.6	18	1.9	312	100.0
Dep3	1146	87.2	210	16.0	21	1.6	798	82.4	210	21.7	21	2.2	372	100.0
Dep4	1419	87.8	240	14.8	30	1.9	951	82.1	240	20.7	30	2.6	501	100.0
Highest quintile	2223	89.7	333	13.4	45	1.8	1536	85.5	333	18.5	45	2.5	726	100.0
**Resident location**														
Urban	5994	88.1	1035	15.2	108	1.6	4212	83.2	1035	20.5	108	2.1	1890	100.0
Rural	1137	87.1	204	15.6	27	2.1	786	81.4	204	21.1	27	2.8	363	100.0

MELAA: Middle Eastern Latin American African

Percentages (%) represent row percentages and may sum to greater than 100% because individuals with an NT-ABI can be identified in more than one dataset

.S: Sample size too small to be reported following data privacy measures

Females had more cases identified in the NMDS and fewer in either ACC or Socrates than males. For age group a greater proportion of identified cases came from the ACC and Socrates datasets for those aged between 5 and 14 years. Proportionally more cases were identified in the NMDS for those aged between 0 and 4 years or between 20 and 24 years. Māori, Pacific, and Asian youth had a greater proportion of cases identified in the NMDS and Socrates datasets than NZ European or MELAA youth. By contrast in the ACC dataset NZ European and MELAA young people had a greater proportion of cases identified from that source.

There were proportionally fewer cases of NT-ABI identified in the ACC dataset as the level of deprivation increased. The opposite trend was evident in the Socrates dataset where more cases were present in that dataset as the level of deprivation increased. When looking at urban vs. rural cases, rural living young people had a lower proportion of cases identified in the NMDS, and a greater proportion identified in the Socrates dataset than urban dwelling young people.

## Discussion

We present a systematic approach to case identification of non-traumatic acquired brain injury in paediatric populations. We compiled ICD-9, ICD-10, and Read codes which were examined by a clinical consensus committee for inclusion in a case definition of non-traumatic brain injury in children. Using multiple datasets contained in the New Zealand Integrated Data Infrastructure, we identified over 8000 cases of NT-ABI among the youth population at a rate of 0.52% in the five year period examined. Our approach makes a significant contribution to NT-ABI research by using multiple classification systems to identify a wide range of potential codes and an expert review process to reach consensus regarding inclusion. In addition, results from an application of the method are presented for ages 0–24 years, including and excluding birth trauma, to account for international differences in classification of acquired brain injury.

Case identification is the foundational step for further research using population data to identify and monitor the healthcare service needs and utilization of clinical populations [28–30]. Future studies could apply this definition to administrative data with the aim of improving the changing needs of people with NT-ABI. Service utilization is high in people living with a non-traumatic brain injury acquired in childhood [20]. Studies within distinct diagnostic groups indicate that education and quality of life sequelae for youth living with a NT-ABI play a significant role in longer-term outcomes across the lifespan [9–11, 31] thereby presenting diverse support needs across health, education, employment, and social services. Monitoring trends in the data for the population of people with NT-ABI as a whole will contribute to the understanding of the unique service needs of this group of people.

A method for identification of NT-ABI allows for the investigation of the costs of ongoing service utilization following non-traumatic injury. Epidemiological measures of the costs associated with NT-ABI require a definition that can be used in population data. Calculation of the years of life lost (YLLs) and years lived with disability (YLDs) can be combined to an estimate of disability adjusted life years (DALYS). Across the age spectrum and a range of causes, neurological disorders carry a significant global burden and in 2016 had the highest cost of DALYs [32]. In children neurological injuries contribute to a high global burden in part due to years lived with disability from a young age [33]. Our results indicate the highest rate of NT-ABI occurs in those aged 0–4 years which is consistent with other reports of high rates of neurological insult and traumatic injury in the preschool years [33, 34]. The effects of early injury on developmental trajectory and on the higher number of years lived with disability contribute to the burden of NT-ABI in children [2, 35]. Burden of injury studies in TBI across all ages suggest high costs associated with injury in the first 12 months and ongoing costs associated with moderate to severe injury [36, 37]. Case identification of moderate to severe NT-ABI in young people could be used to estimate costs associated with persistent disability across the sub-groups of brain injury and identify the costs associated with specific service use.

We aimed to provide a method of case identification for NT-ABI in young people using national administrative data but the method was not designed with the intention of estimating prevalence. Several authors have discussed the limitations of administrative data for prevalence estimates and warned against using such estimates for funding decisions [13, 14]. Prevalence estimates from population-level administrative data are generally accepted to underestimate the brain injury rate substantially. In a validation study of the application of ICD-10 codes to a cohort of cases known to have TBI, only 18% of known TBI cases had appropriate ICD10 codes in administrative data [15]. Studies comparing TBI codes present in hospital level administrative data with medical records indicate that the rate of TBI is underestimated [38–40]. Codes for skull fractures, intracranial lesions, and neurosurgical procedures may be more reliably present in hospital level data [40] while neurological conditions may be underestimated [39]. Internal neurological events leading to NT-ABI are therefore also likely to be underestimated in the hospital level data that feeds into population level datasets such as the IDI. A limitation of our method of case identification is that it is currently unvalidated and therefore the degree of undercount or false positives is unknown. Future studies using medical records to examine the application of appropriate NT-ABI codes in the youth population from medical records through to administrative data would assist in validating case identification. This would allow an iterative approach to refine this method of case identification.

The five-year rate of NT-ABI in our cohort will significantly underestimate the true rate. We used stringent criteria for identifying NT-ABI cases and included only codes where a brain injury was specified in the code or considered likely to have resulted in a brain injury according to the clinical consensus committee. There is no classification system for the severity of NT-ABI, and mild NT-ABI is likely undetected in hospital records that feed into administrative data. Therefore, the NT-ABI cases identified will represent only a proportion of severe and moderately severe NT-ABI cases. Clear severity classification guidelines exist for traumatic injuries [41]. Even with this system, it is well documented that mild traumatic injuries are more likely to be absent from administrative data [15]. To provide incidence estimates requires identification of the first time an NT-ABI code appears for a young person. This necessitates the availability of robust data with time coverage that extends beyond that currently present in the IDI. Our approach examined the rate of codes present in a five year period but does not provide information about the timing of injury. Calculation of incidence estimates is an important focus for future research as more data become available.

We used a clinical consensus approach similar to that used in the identification of youth mental health problems [21]. The clinical consensus committee that reviewed codes for inclusion in a definition of NT-ABI was comprised of clinicians with experience across a wide range of NT-ABI presentations. In New Zealand there is a single Pediatric Rehabilitation Service that provides specialist rehabilitation to children, therefore clinicians working in this service are familiar with the spectrum of causes of NT-ABI. However, a consensus committee comprised of experts across individual causes of neurological insult may have arrived at a different set of codes for inclusion. In depth knowledge of neurological conditions and their functional outcome over time is required to understand NT-ABI. Future studies could bring together clinicians with specific expertise in sub-groups of neurological conditions affecting children in an effort to determine codes for inclusion and to compare these with those identified here. Other approaches to case identification have also been reported. In their study of TBI, Chen and Colantonio (2011) used a systematic review of the literature to examine the use of ICD-10 codes for TBI. They identified 26 studies and noted a high degree of inconsistency between studies in use of ICD-10 codes [18]. The challenges in applying a systematic literature review approach to NT-ABI include the diverse range of neurological conditions that can cause NT-ABI. Decisions about which conditions to include in a literature search would still need to be informed by clinical knowledge of NT-ABI rehabilitation and ideally by consensus. Nevertheless, a systematic literature review would provide an alternative approach to case identification that could be compared to the clinical consensus method. An advantage of our method is the review of ICD-9, ICD-10, and Read codes to capture NT-ABI in administrative data that spans timeframes during which classification systems can change.

We found a clear socioeconomic gradient of case identification of non-traumatic ABI. Cases of NT-ABI were higher in young people living in the most economically deprived households. This gradient was present in birth trauma cases and NT-ABI cases with birth trauma excluded. Socioeconomic deprivation is a documented risk factor for traumatic brain injury [42–44]. It is unclear why non-traumatic brain injuries occurring as the result of internal neurological processes are more common in high-deprivation households. Barriers to healthcare access and factors associated with deprivation may lead to circumstances where brain insults are more likely to occur for example increased childhood infections. Higher levels of socioeconomic deprivation in early childhood have been found to increase the risk for multimorbidity and chronic illness [45]. Socioeconomic factors represent a potentially modifiable risk factor that could reduce rates of NT-ABI in childhood. For traumatic brain injuries, prevention efforts have focused on reducing injury from road traffic accidents, falls, and sports injuries. This work demonstrates the possibilities for injury prevention that emerge from an understanding of modifiable risk factors. Our finding highlights the need to investigate the potential reasons for this deprivation gradient in future studies.

Most NT-ABI cases were identified from the NMDS dataset (hospital inpatient records), with birth trauma cases exclusively from this dataset. Given that children with medical events or diagnoses resulting in a non-traumatic brain injury are treated in a hospital setting, it is unsurprising that most cases were identified through the NMDS. No cases of NT-ABI came from the PRIMHD (community mental health) dataset. Although acquired brain injury is a risk factor for mental health problems [46], PRIMHD reflects tertiary-level mental health service use where mental health diagnoses are the focus. Physical health diagnoses underlying a NT-ABI would not necessarily appear in these services, and the lack of case identification through PRIMHD is not unexpected. Future studies using the IDI to explore NT-ABI could investigate mental health outcomes using PRIMHD rather than using this dataset to identify cases of NT-ABI. ACC (accident claims) and Socrates (disability support services) contributed a relatively small number of NT-ABI cases but did contribute to case identification. Previously, researchers have found that utilization of multiple databases in the IDI is preferable to capture cases more thoroughly [21]. Our findings suggest that using multiple datasets to identify cases of NT-ABI will result in better capture of cases, particularly for brain injuries occurring after the perinatal period.

## Conclusions

To provide timely and relevant healthcare intervention, researchers need to understand the rate of occurrence as well as the changing needs and trajectories of young people living with an NT-ABI. We provide a method for identifying cases of NT-ABI in population level administrative data and a framework for working with multiple datasets. A method of case identification is the first step toward monitoring trends in the population of young people with NT-ABI as a whole, understanding service needs, targeting prevention, and ultimately providing coordinated, consistent care to people living with a non-traumatic brain injury acquired in childhood.

### Electronic supplementary material

Below is the link to the electronic supplementary material.

Supplementary Material 1

## Data Availability

The data used in this study are held with the Integrated Data Infrastructure and are managed by Statistics New Zealand. These data are publicly available, although access to them is restricted. Please see https://www.stats.govt.nz/integrated-data/integrated-data-infrastructure/ for more details. The SAS code will be made available to interested parties.

## Abbreviations

ABI: Acquired Brain Injury
ACC: Accident Compensation Corporation
DSM: Diagnostic and Statistical Manual for Mental Disorders
ERP: Estimated resident population
ICD: International Classification of Diseases
IDI: Integrated Data Infrastructure
MELAA: Middle Eastern, Latin American, African
NMDS: National Minimum Dataset
NT-ABI: Non-traumatic acquired brain injury
PRIMHD: Programme for the Integration of Mental Health Data
TBI: Traumatic brain injury

## References

[1] Camm S, Porter M, Brooks A, Boulton K, Veloso GC (2021). Cognitive interventions for children with acquired brain injury: a systematic review. Neuropsychological Rehabilitation.

[2] Anderson V, Spencer-Smith M, Wood A (2011). Do children really recover better? Neurobehavioural plasticity after early brain insult. Brain.

[3] Hooper SR, Alexander J, Moore D, Sasser HC, Laurent S, King J (2004). Caregiver reports of common symptoms in children following a traumatic brain injury. NeuroRehabilitation.

[4] Ylvisaker M, Turkstra LS, Coelho C, editors. Behavioral and social interventions for individuals with traumatic brain injury: A summary of the research with clinical implications. Seminars in speech and language; 2005: Copyright© 2005 by Thieme Medical Publishers, Inc., 333 Seventh Avenue, New &#8230. 10.1055/s-2005-922104 16278797

[5] Anderson V, Northam E, Wrennall J. Developmental neuropsychology: a clinical approach. Routledge; 2018.

[6] Cullen NK, Park Y-G, Bayley MT (2008). Functional recovery following traumatic vs non-traumatic brain injury: a case-controlled study. Brain Injury.

[7] Smania N, Avesani R, Roncari L, Ianes P, Girardi P, Varalta V (2013). Factors predicting functional and cognitive recovery following severe traumatic, anoxic, and cerebrovascular brain damage. J Head Trauma Rehabil.

[8] Rajesh A, Daugherty AM, Jain S, Henry D, Barbey AK, Rubin RD (2020). Comorbid conditions differentiate rehabilitation profiles in traumatic versus nontraumatic brain injury: a retrospective analysis using a medical database. J Head Trauma Rehabilitation.

[9] Annett RD, Patel SK, Phipps S (2015). Monitoring and assessment of neuropsychological outcomes as a standard of care in pediatric oncology. Pediatr Blood Cancer.

[10] Fowler Å, Stödberg T, Eriksson M, Wickström R (2010). Long-term outcomes of acute encephalitis in childhood. Pediatrics.

[11] Donoghue AJ, Nadkarni V, Berg RA, Osmond MH, Wells G, Nesbitt L (2005). Out-of-hospital pediatric cardiac arrest: an epidemiologic review and assessment of current knowledge. Ann Emerg Med.

[12] Biermans MC, Verheij RA, de Bakker DH, Zielhuis GA, de Vries Robbé PF (2008). Estimating morbidity Ratesfrom Electronic Medical Records in General Practice. Methods Inf Med.

[13] Roozenbeek B, Maas AI, Menon DK (2013). Changing patterns in the epidemiology of traumatic brain injury. Nat Reviews Neurol.

[14] Chan V, Thurairajah P, Colantonio A (2015). Defining pediatric traumatic brain injury using international classification of diseases Version 10 codes: a systematic review. BMC Neurol.

[15] Barker-Collo S, Theadom A, Jones K, Feigin VL, Kahan M (2016). Accuracy of an international classification of diseases code surveillance system in the identification of traumatic brain injury. Neuroepidemiology.

[16] Warwick J, Slavova S, Bush J, Costich J (2020). Validation of ICD-10-CM surveillance codes for traumatic brain injury inpatient hospitalizations. Brain Injury.

[17] McChesney-Corbeil J, Barlow K, Quan H, Chen G, Wiebe S, Jette N (2017). Validation of a case definition for pediatric brain injury using administrative data. Can J Neurol Sci.

[18] Chen AY, Colantonio A (2011). Defining neurotrauma in administrative data using the International classification of diseases Tenth Revision. Emerg Themes Epidemiol.

[19] Amy C, Zagorski B, Chan V, Parsons D, Vander Laan R, Colantonio A (2012). Acute care alternate-level-of-care days due to delayed discharge for traumatic and non-traumatic brain injuries. Healthc Policy.

[20] Chan V, Pole JD, Keightley M, Mann RE, Colantonio A (2016). Children and youth with non-traumatic brain injury: a population based perspective. BMC Neurol.

[21] Bowden N, Gibb S, Thabrew H, Kokaua J, Audas R, Merry S (2020). Case identification of mental health and related problems in children and young people using the New Zealand Integrated Data infrastructure. BMC Med Inf Decis Mak.

[22] Kvalsvig A, Gibb S, Teng A. Linkage error and linkage bias: a guide for IDI users. University of Otago; 2019. https://vhin.co.nz/wp-content/uploads/2019/11/Linkage-error-and-linkage-bias.pdf.

[23] NZ. S. Integrated Data Infrastructure. 2023. https://www.stats.govt.nz/integrated-data/integrated-data-infrastructure/.

[24] Booth N (1994). What are the read codes?. Health Libr Rev.

[25] NZ S. 2018 Census Ethnic Group Summaries. 2023. https://www.stats.govt.nz/tools/2018-census-ethnic-group-summaries/.

[26] Zhao J, Gibb S, Jackson R, Mehta S, Exeter DJ. Aust N Z J Public Health. 2018;42(4):382–8. Constructing whole of population cohorts for health and social research using the New Zealand Integrated Data Infrastructure. 10.1111/1753-6405.12781 29644776

[27] Gibb S, Bycroft C, Matheson-Dunning N. Identifying the New Zealand resident population in the integrated data infrastructure (IDI). Statistics New Zealand = Tatauranga Aotearoa; 2016.

[28] Bowden N, Gibb S, Audas R, Clendon S, Dacombe J, Kokaua J (2022). Association between high-need education-based funding and school suspension rates for autistic students in New Zealand. JAMA Pediatr.

[29] Mujoo H, Bowden N, Thabrew H, Kokaua J, Audas R, Taylor B. Identifying neurodevelopmental disabilities from nationalised preschool health check. Australian New Z J Psychiatry. 2023:00048674231151606. 10.1177/00048674231151606 PMC10363952 36748102

[30] Richmond-Rakerd LS, D’Souza S, Milne BJ, Caspi A, Moffitt TE (2022). Longitudinal associations of mental disorders with dementia: 30-year analysis of 1.7 million New Zealand citizens. JAMA Psychiatry.

[31] Welbourn C, Efstathiou N (2018). How does the length of cardiopulmonary resuscitation affect brain damage in patients surviving cardiac arrest? A systematic review. Scand J Trauma Resusc Emerg Med.

[32] Feigin VL, Nichols E, Alam T, Bannick MS, Beghi E, Blake N (2019). Global, regional, and national burden of neurological disorders, 1990–2016: a systematic analysis for the global burden of Disease Study 2016. Lancet Neurol.

[33] Newton CR, editor. Editor global burden of pediatric neurological disorders. Seminars in pediatric neurology. Elsevier; 2018. 10.1016/j.spen.2018.03.002 PMC7613506 30293585

[34] Theadom A, Barker-Collo S, Feigin VL, Starkey NJ, Jones K, Jones A (2012). The spectrum captured: a methodological approach to studying incidence and outcomes of traumatic brain injury on a population level. Neuroepidemiology.

[35] Alli SR, Gorbovskaya I, Liu JC, Kolla NJ, Brown L, Müller DJ (2022). The gut microbiome in depression and potential benefit of prebiotics, probiotics and synbiotics: a systematic review of clinical trials and observational studies. Int J Mol Sci.

[36] Te Ao B, Brown P, Tobias M, Ameratunga S, Barker-Collo S, Theadom A (2014). Cost of traumatic brain injury in New Zealand: evidence from a population-based study. Neurology.

[37] Te Ao B, Tobias M, Ameratunga S, McPherson K, Theadom A, Dowell A (2015). Burden of traumatic brain injury in New Zealand: incidence, prevalence and disability-adjusted life years. Neuroepidemiology.

[38] DEB S (1999). ICD-10 codes detect only a proportion of all head injury admissions. Brain Injury.

[39] Shore AD, Mccarthy ML, Serpi T, Gertner M (2005). Validity of administrative data for characterizing traumatic brain injury-related hospitalizations. Brain Injury.

[40] Carroll CP, Cochran JA, Guse CE, Wang MC (2012). Are we underestimating the burden of traumatic brain injury? Surveillance of severe traumatic brain injury using centers for disease control international classification of disease, ninth revision, clinical modification, traumatic brain injury codes. Neurosurgery.

[41] Hawryluk GW, Manley GT (2015). Classification of traumatic brain injury: past, present, and future. Handb Clin Neurol.

[42] Amram O, Schuurman N, Pike I, Yanchar NL, Friger M, McBeth PB (2015). Socio economic status and traumatic brain injury amongst pediatric populations: a spatial analysis in greater Vancouver. Int J Environ Res Public Health.

[43] Faelker T, Pickett W, Brison RJ (2000). Socioeconomic differences in childhood injury: a population based epidemiologic study in Ontario, Canada. Inj Prev.

[44] Pozzato I, Tate RL, Rosenkoetter U, Cameron ID (2019). Epidemiology of hospitalised traumatic brain injury in the state of New South Wales, Australia: a population-based study. Aust N Z J Public Health.

[45] Russell J, Grant CC, Morton SM (2020). Multimorbidity in early childhood and socioeconomic disadvantage: findings from a large New Zealand child cohort. Acad Pediatr.

[46] McKinlay A, Grace R, Horwood J, Fergusson D, MacFarlane M (2009). Adolescent psychiatric symptoms following preschool childhood mild traumatic brain injury: evidence from a birth cohort. J Head Trauma Rehabil.

