# Supplementary material for: Case identification of non-traumatic brain injury in youth using linked population data
Source: BMC Neurol. 2024 Mar 2;24:82. doi: 10.1186/s12883-024-03575-6 (PMC10908152; doi:10.1186/s12883-024-03575-6)
Supplement: Supplementary file 1 — Supplementary Material 1 [file 12883_2024_3575_MOESM1_ESM.docx]

**Table S1: Case identification codes for non-traumatic acquired brain injury from ICD-9CM and ICD010AM**

| **ICD-9CM Aii** |  | **ICD-10 AM** |
| --- | --- | --- |
| 3481 (anoxic brain damage) |  | G931 (Anoxic brain damage) (includes all causes of anoxia except those occurring following abortions, ectopic pregnancy, labour and delivery and newborn) |
| 9947 (asphyxiation and strangulation) |  | T71 (Asphyxiation, suffocation) (by strangulation) |
| 9941 (drowning and nonfatal submersion) |  | T751 (Drowning and nonfatal submersion) |
| 7990 (asphyxia) |  | R090 (Asphyxia) |
| 430 (subarachnoid haemorrhage) |  | I600 (Subarachnoid haemorrhage from carotid siphon and bifurcation) |
| 431 (intracerebral haemorrhage) |  | I601 (Subarachnoid haemorrhage from middle cerebral artery) |
| 4320 (nontraumatic extradural haemorrhage) |  | I602 (Subarachnoid haemorrhage from anterior communicating artery) |
| 4321 (subdural haemorrhage, nontraumatic) |  | I603 (Subarachnoid haemorrhage from posterior communicating artery) |
| 4329 (unspecified intracranial haemorrhage) |  | I613 (Intracerebral haemorrhage in brain stem) |
| 43301 (basilar artery with cerebral infarction) |  | I615 (Intracerebral haemorrhage, intraventricular) |
| 43311 (carotid artery with cerebral infarction) |  | I618 (Other intracerebral haemorrhage) |
| 43321 (vertebral artery with cerebral infarction) |  | I620 (Subdural haemorrhage) (acute) (nontraumatic) |
| 43331 (multiple and bilateral with cerebral infarction) |  | I629 (Intracranial haemorrhage, unspecified) (nontraumatic) |
| 43381 (other specified precerebral artery with cerebral infarction) |  | I631 (Cerebral infarction due to embolism of precerebral arteries) |
| 43391 (unspecified precerebral artery with cerebral infarction) |  | I633 (Cerebral infarction due to thrombosis of cerebral arteries) |
| 43401 (cerebral thrombosis with cerebral infarction) |  | I635 (Cerebral infarction due to unspecified occlusion or stenosis of cerebral arteries) |
| 43411 (cerebral embolism with cerebral infarction) |  | I638 (Other cerebral infarction) |
| 43491 (cerebral artery occlusion, unspecified with cerebral infarction) |  | I694 (Sequelae of stroke, not specified as haemorrhage or infarction) (Sequelae of cerebrovascular disease) |
| 436 (acute, but ill-defined, cerebrovascular disease) |  | B010 (Varicella meningitis) |
| 4371 (other generalised ischaemic cerebrovascular disease) |  | I604 (Subarachnoid haemorrhage from basilar artery) |
| 4372 (hypertensive encephalopathy) |  | I605 (Subarachnoid haemorrhage from vertebral artery) |
| 4374 (cerebral arteritis) |  | I606 (Subarachnoid haemorrhage from other intracranial arteries) |
| 4375 (Moya Moya disease) |  | I607(Subarachnoid haemorrhage from intracranial artery, unspecified) |
| 4376 (nonpyrogenic thrombosis of intracranial venous sinus) |  | I608 (Other subarachnoid haemorrhage) |
| 438 (late effects of cerebrovascular disease) |  | I609 (Subarachnoid haemorrhage, unspecified) |
| 1910 (malignant neoplasm of brain) (cerebrum, except lobes and ventricles) |  | I610 (Intracerebral haemorrhage in hemisphere, subcortical) |
| 1911 (malignant neoplasm of brain) (frontal lobe) |  | I611 (Intracerebral haemorrhage in hemisphere, cortical) |
| 1912 (malignant neoplasm of brain) (temporal lobe) |  | I612 (Intracerebral haemorrhage in hemisphere, unspecified) |
| 1913 (malignant neoplasm of brain) (parietal lobe) |  | I614 (Intracerebral haemorrhage in cerebellum) |
| 1914 (malignant neoplasm of brain) (parietal lobe) |  | I616 (Intracerebral haemorrhage, multiple localised) |
| 1915 (malignant neoplasm of brain) (ventricles) |  | I619 (Intracerebral haemorrhage, unspecified) |
| 1916 (malignant neoplasm of brain) (cerebellum NOS) |  | I621 (Nontraumatic extradural haemorrhage) |
| 1917 (malignant neoplasm of brain) (brain stem) |  | I630 (Cerebral infarction due to thrombosis of precerebral arteries) |
| 1918 (malignant neoplasm of brain) (other parts of brain) |  | I632 (Cerebral infarction due to unspecified occlusion or stenosis of precerebral arteries) |
| 1919 (malignant neoplasm of brain) (brain, unspecified) |  | I634 (Cerebral infarction due to embolism of cerebral arteries) |
| 1920 (malignant neoplasm of cranial nerves) |  | I636 (Cerebral infarction due to cerebral venous thrombosis, nonpyogenic) |
| 1921 (malignant neoplasm of cerebral meninges) |  | I639 (Cerebral infarction, unspecified) |
| 1983 (secondary malignant neoplasm of other specified sites, brain and spinal cord) |  | I64 (Stroke, not specified as haemorrhage or infarction) |
| 1984 (secondary malignant neoplasm of other specified sites, meninges cerebral/spinal) |  | I672 (Cerebral atherosclerosis) (Other cerebrovascular diseases) |
| 2250 (benign neoplasm of brain and other parts of nervous system, brain) |  | I673 (Progressive vascular leukoencephalopathy) (Other cerebrovascular diseases) |
| 2251 (benign neoplasm of brain and other parts of nervous system, cranial nerves) |  | I674 (Hypertensive encephalopathy) (Other cerebrovascular diseases) |
| 2252 (benign neoplasm of brain and other parts of nervous system, cerebral meninges) |  | I675 (Moya Moya disease) (Other cerebrovascular diseases) |
| 2375 (neoplasm of uncertain behaviour of endocrine glands and nervous system, brain and spinal cord) |  | I676 (Nonpyogenic thrombosis of intracranial venous system) (Other cerebrovascular diseases) |
| 3240 (intracranial abscess) |  | I677 (Cerebral arteritis, not elsewhere classified) (Other cerebrovascular diseases) |
| 3249 (extradural or subdural abscess NOS) |  | I678 (Other specified cerebrovascular diseases) (Other cerebrovascular diseases) |
| 3480 (cerebral cysts) |  | I679 (Other specified cerebrovascular diseases) (Other cerebrovascular diseases) |
| 2503 (diabetes with other coma) |  | I680 (Cerebral amyloid angiopathy) (Cerebrovascular disorders in diseases classified elsewhere) |
| 2510 (hypoglycaemic coma) |  | I681 (Cerebral arteritis in infectious and parasitic diseases classified elsewhere) (Cerebrovascular disorders in diseases classified elsewhere) |
| 3200 (haemophilus meningitis) |  | I682 (Cerebral arteritis in other diseases classified elsewhere) (Cerebrovascular disorders in diseases classified elsewhere) |
| 3201 (pneumococcal meningitis) |  | I688 (Other cerebrovascular disorders in diseases classified elsewhere) (Cerebrovascular disorders in diseases classified elsewhere) |
| 3202 (streptococcal meningitis) |  | I690 (Sequelae of subarachnoid haemorrhage) (Sequelae of cerebrovascular disease) |
| 3203 (staphylococcal meningitis) |  | I691 (Sequelae of intracerebral haemorrhage) (Sequelae of cerebrovascular disease) |
| 3207 (meningitis in other bacterial diseases classified elsewhere) |  | I692 (Sequelae of other non traumatic intracranial haemorrhage) (Sequelae of cerebrovascular disease) |
| 3208 (meningitis due to other specified bacteria) |  | I693 (Sequelae of cerebral infarction) (Sequelae of cerebrovascular disease) |
| 32081 (anaerobic meningitis) |  | I698 (Sequelae of other and unspecified cerebrovascular diseases) (Sequelae of cerebrovascular disease) |
| 32082 (meningitis due to gram negative bacteria, NOS |  | C700 (Malignant neoplasm of cerebral meninges) |
| 32089 (meningitis due to other specified bacteria) |  | C710 (Malignant neoplasm of cerebrum, except lobes and ventricles) |
| 3209 (meningitis due to unspecified bacteria) |  | C711 (Malignant neoplasm of frontal lobe) |
| 3210 (cryptococcal meningitis) |  | C712 (Malignant neoplasm of temporal lobe) |
| 3211 (meningitis in other fungal diseases) |  | C713 (Malignant neoplasm of parietal lobe) |
| 3212 (meningitis due to viruses not elsewhere classified) |  | C714 (Malignant neoplasm of occipital lobe) |
| 3213 (meningitis due to trypanosomiasis) |  | C715 (Malignant neoplasm of cerebral ventricle) |
| 3214 (meningitis in sarcoidosis) |  | C716 (Malignant neoplasm of cerebellum) |
| 3218 (meningitis due to other nonbacterial organisms classified elsewhere) |  | C717 (Malignant neoplasm of brain stem) |
| 3220 (nonpyogenic meningitis) |  | C718 (Malignant neoplasm of overlapping lesion of brain) |
| 3221 (eosinophilic meningitis) |  | C719 (Malignant neoplasm of brain, unspecified) |
| 3222 (chronic meningitis) |  | C793 (Secondary malignant neoplasm of brain and cerebral meninges) |
| 3229 (meningitis unspecified) |  | C794 (Secondary malignant neoplasm of other and unspecified part of nervous system) |
| 3230 (encephalitis in viral diseases classified elsewhere) |  | D320 (Benign neoplasm of cerebral meninges) |
| 3231 (encephalitis in rickettsial diseases classified elsewhere) |  | D330 (Benign neoplasm of brain, supratentorial) |
| 3232 (encephalitis in protozoal diseases classified elsewhere) |  | D331 (Benign neoplasm of brain, infratentorial) |
| 3234 (other encephalitis due to infection classified elsewhere) |  | D332 (Benign neoplasm of brain, unspecified) |
| 3235 (encephalitis following immunisation procedures) |  | D333 (Benign neoplasm of cranial nerves) |
| 3236 (post infectious meningitis) |  | D420 (Neoplasm of uncertain or unknown behaviour of cerebral meninges) |
| 3237 (toxic encephalitis) |  | D430 (Brain, supratentorial) (Neoplasm of uncertain or unknown behaviour of brain and central nervous system) |
| 3238 (other causes of encephalitis) |  | D431 (Brain, infratentorial) (Neoplasm of uncertain or unknown behaviour of brain and central nervous system) |
| 3239 (unspecified cause of encephalitis) |  | D432 (Brain, unspecified) (Neoplasm of uncertain or unknown behaviour of brain and central nervous system) |
| 0360 (meningococcal meningitis) |  | D433 (Cranial nerves) (Neoplasm of uncertain or unknown behaviour of brain and central nervous system) |
| 0361 (meningococcal encephalitis) |  | G060 (Intracranial abscess and granuloma) |
| 0362 (meningococcaemia) |  | G062 (Extradural and subdural abscess, unspecified) |
| 0369 (meningococcal infection, unspecified) |  | G07 (Intracranial and intraspinal abscess and granuloma in disease classified elsewhere) |
| 01300 (tuberculous meningitis) |  | G930 (Cerebral cysts) |
| 0131 (tuberculoma of meninges) |  | E1002 (Type I) with coma |
| 0132 (tuberculoma of brain) |  | E1102 (Type II) with coma |
| 0133 (tuberculous abscess of brain) |  | E1302 (Other specified diabetes mellitus with coma) |
| 0620 (Japanese encephalitis) |  | E1402 (Unspecified diabetes mellitus with coma) |
| 0621 (western equine encephalitis) |  | E15 (Nondiabetic hypoglycaemic coma) |
| 0622 (eastern equine encephalitis) |  | G92 (Toxic encephalopathy) |
| 0623 (st. louis encephalitis) |  | G934 (Encephalopathy, unspecified) |
| 0624 ( Australian encephalitis) |  | A390 (Meningococcal meningitis) |
| 0625 (California virus encephalitis) |  | A392 (Acute meningococcaemia) |
| 0628 (other specified mosquito-borne viral encephalitis) |  | A393 (Chronic meningococcaemia) |
| 0629 (mosquito-borne viral encephalitis unspecified) |  | A394 (Meningococcaemia) |
| 0630 (Russian spring-summer encephalitis) |  | A398 (Other meningococcal infections) |
| 0631 (louping ill) |  | A399 (Meningococcal infection, unspecified) |
| 0632 (central European encephalitis) |  | A170 (Meningeal meningitis) |
| 0638 (other specified tick-borne viral encephalitis) |  | A171 (Meningeal tuberculoma) |
| 0639 (tick-borne viral encephalitis unspecified) |  | A321 (Listerial meningitis and meningoencephalitis) |
| 064 (viral encephalitis transmitted by other and unspecified arthropods) |  | A83 (Mosquito-borne viral encephalitis) |
| 0470 (coxsackie virus) |  | A830 (Japanese encephalitis) |
| 0471 (echo virus) |  | A831 (Western equine encephalitis) |
| 0478 (other specified viral meningitis) |  | A832 (Eastern equine encephalitis) |
| 0479 (unspecified viral meningitis) |  | A833 (St Louis encephalitis) |
| 0490 (lymphocytic choriomeningitis) |  | A834 (Australian encephalitis) |
| 0491 (meningitis due to adenovirus) |  | A835 (California encephalitis) |
| 0498 (other specified non arthropod borne viral diseases of central nervous system) |  | A836 (Rocio virus disease) |
| 0499 (viral encephalitis) |  | A838 (Other mosquito-borne viral encephalitis) |
| 0462 (subacute sclerosing panencephalitis) |  | A839 (Mosquito-borne viral encephalitis) |
| 0520 (post varicella encephalitis) |  | A840 (Far eastern tick-borne encephalitis) (Tick-borne viral encephalitis) |
| 0530 (herpes zoster with meningitis) |  | A841 (Central European tick-borne encephalitis) (Tick-borne viral encephalitis) |
| 05472 (herpes simplex meningitis) |  | A848 (Other tick-borne viral encephalitis) (Tick-borne viral encephalitis) |
| 0543 (herpes meningoencephalitis) |  | A849 (Tick-borne viral encephalitis, unspecified ) (Tick-borne viral encephalitis) |
| 0550 (post measles encephalitis) |  | A850 (Enteroviral encephalitis) (Other viral encephalitis, not elsewhere classified) |
| 0721 (mumps meningitis) |  | A851 (Adenoviral encephalitis) (Other viral encephalitis, not elsewhere classified) |
| 0722 (mumps encephalitis) |  | A852 (Arthropod-borne viral encephalitis, unspecified) (Other viral encephalitis, not elsewhere classified) |
| 11283 (candidal meningitis) |  | A858 (Other specified viral encephalitis) (Other viral encephalitis, not elsewhere classified) |
| 3108 (other specified nonpsychotic mental disorders following organic brain damage) (postencephalitic syndrome) |  | A86 (Unspecified viral encephalitis) |
| 1390 (late effects of viral encephalitis) |  | A870 (Enteroviral meningitis) (Viral meningitis) |
| 326 (late effects of intracranial abscess of pyogenic infection) |  | A871 (Adenoviral meningitis) (Viral meningitis) |
| 3313 (communicating hydrocephalus) |  | A872 (Lymphocytic meningitis) (Viral meningitis) |
| 3314 (obstructive hydrocephalus) |  | A878 (Other viral meningitis) (Viral meningitis) |
| 3483 (encephalopathy, unspecified) |  | A879 (Viral meningitis, unspecified) (Viral meningitis) |
| 3484 (compression of brain) |  | A811 (Subacute, sclerosing encephalitis) |
| 3485 (cerebral oedema) |  | B003 (Herpes viral meningitis) |
| 3488 (other conditions of brain) |  | B004 (Herpes viral meningoencephalitis) |
| 3489 (unspecified condition of brain) |  | B011 (Varicella encephalitis) |
| 7816 (meningism’s) |  | B020 (Zoster encephalitis) |
|  |  | B021 (Zoster meningitis) |
|  |  | B050 (Post measles encephalitis) |
|  |  | B051 (Post measles meningitis) |
|  |  | B261 (Mumps meningitis) |
|  |  | B262 (Mumps encephalitis) |
|  |  | B375 (Candidal meningitis) |
|  |  | B451 (Cerebral cryptococcosis) |
|  |  | F071 (Post encephalitic syndrome) |
|  |  | B941 (Sequelae of viral encephalitis) |
|  |  | G040 (Acute disseminated encephalitis) |
|  |  | G042 (Bacterial meningoencephalitis and meningomyelitis, not elsewhere classified) |
|  |  | G048 (Other encephalitis, myelitis and encephalomyelitis) |
|  |  | G049 (Encephalitis, myelitis, and encephalomyelitis, unspecified) |
|  |  | G05 (Encephalitis, myelitis, and encephalomyelitis in diseases classified elsewhere) |
|  |  | G09 (Sequelae of inflammatory diseases of central nervous system) |
|  |  | G000 (Bacterial meningitis, not elsewhere classified) (haemophilus meningitis) |
|  |  | G001 (Bacterial meningitis, not elsewhere classified) (pneumococcal meningitis) |
|  |  | G002 (Bacterial meningitis, not elsewhere classified) (streptococcal meningitis) |
|  |  | G003 (Bacterial meningitis, not elsewhere classified) (staphylococcal meningitis) |
|  |  | G008 (Bacterial meningitis, not elsewhere classified) (other bacterial meningitis) |
|  |  | G009 (Bacterial meningitis, not elsewhere classified) (bacterial meningitis, unspecified) |
|  |  | G050 (Encephalitis, myelitis, and encephalomyelitis in bacterial diseases classified elsewhere) |
|  |  | G051 (Encephalitis, myelitis, and encephalomyelitis in viral diseases classified elsewhere) |
|  |  | G052 (Encephalitis, myelitis, and encephalomyelitis in other infectious and parasitic diseases classified elsewhere) |
|  |  | G058 (Encephalitis, myelitis, and encephalomyelitis in other diseases classified elsewhere) |
|  |  | G020 (Meningitis in viral diseases classified elsewhere) classified elsewhere) |
|  |  | G021 (Meningitis in mycoses) |
|  |  | G028 (Meningitis in other specified infectious and parasitic diseases classified elsewhere) |
|  |  | G030 (Nonpyogenic meningitis) |
|  |  | G031 (Chronic meningitis) |
|  |  | G032 (Benign recurrent meningitis) |
|  |  | G038 (Meningitis due to other specified causes) |
|  |  | G039 (Meningitis, unspecified) |
|  |  | G040 (Acute disseminated encephalitis) |
|  |  | G042 (Bacterial meningoencephalitis and meningomyelitis, not elsewhere classified) |
|  |  | G048 (Other encephalitis, myelitis and encephalomyelitis) |
|  |  | G049 (Encephalitis, myelitis, and encephalomyelitis, unspecified) |
|  |  | G050 (Encephalitis, myelitis and encephalomyelitis in diseases classified elsewhere) |
|  |  | G051 (Encephalitis, myelitis and encephalomyelitis in viral diseases classified elsewhere) |
|  |  | G052 (Encephalitis, myelitis and encephalomyelitis in other infectious and parasitic diseases classified elsewhere) |
|  |  | G058 (Encephalitis, myelitis and encephalomyelitis in other diseases classified elsewhere) |
|  |  | G910 (Communicating hydrocephalus) |
|  |  | G911 (Obstructive hydrocephalus) |
|  |  | G935 (Compression of brain) |
|  |  | G936 (Cerebral oedema) |
|  |  | G938 (Other specified disorders of the brain) (including post radiation encephalopathy) |
|  |  | G939 (Disorder of the brain, unspecified) |
|  |  | R291 (Meningism us) |

**Table S2: List of ICD-9CM and ICD-10AM birth trauma codes for case identification**

| **Birth Trauma Codes** | | |
| --- | --- | --- |
| **ICD-9CM Aii** |  | **ICD-10 AM** |
| P10.0 Subdural haemorrhage due to birth injury |  | P10.0 Subdural haemorrhage due to birth injury |
| P10.1 Cerebral haemorrhage due to birth injury |  | P10.1 Cerebral haemorrhage due to birth injury |
| P10.2 Intraventricular haemorrhage due to birth injury |  | P10.2 Intraventricular haemorrhage due to birth injury |
| P10.3 Subarachnoid haemorrhage due to birth injury |  | P10.3 Subarachnoid haemorrhage due to birth injury |
| P10.4 Tentorial tear due to birth injury |  | P10.4 Tentorial tear due to birth injury |
| P10.8 Other intracranial lacerations and haemorrhages due to birth injury |  | P10.8 Other intracranial lacerations and haemorrhages due to birth injury |
| P10.9 Unspecified intracranial laceration and haemorrhage due to birth injury |  | P10.9 Unspecified intracranial laceration and haemorrhage due to birth injury |
| P11.0 Cerebral oedema due to birth injury |  | P11.0 Cerebral oedema due to birth injury |
| P11.1 Other specified brain damage due to birth injury |  | P11.1 Other specified brain damage due to birth injury |
| P11.2 Unspecified brain damage due to birth injury |  | P11.2 Unspecified brain damage due to birth injury |
| P21.0 Severe birth asphyxia |  | P21.0 Severe birth asphyxia |
| P21.1 Mild and moderate birth asphyxia |  | P21.1 Mild and moderate birth asphyxia |
| P21.9 Birth asphyxia, unspecified |  | P21.9 Birth asphyxia, unspecified |
| P52.0 Intraventricular (nontraumatic) haemorrhage, grade 1, of foetus and newborn (Subependymal haemorrhage (without intraventricular extension) |  | P52.0 Intraventricular (nontraumatic) haemorrhage, grade 1, of fetus and newborn (Subependymal haemorrhage (without intraventricular extension) |
| P52.1Intraventricular (nontraumatic) haemorrhage, grade 2, of fetus and newborn |  | P52.1Intraventricular (nontraumatic) haemorrhage, grade 2, of fetus and newborn |
| P52.2Intraventricular (nontraumatic) haemorrhage, grade 3, and grade 4 of fetus and newborn |  | P52.2Intraventricular (nontraumatic) haemorrhage, grade 3, and grade 4 of fetus and newborn |
| P52.3Unspecified intraventricular (nontraumatic) haemorrhage of fetus and newborn |  | P52.3Unspecified intraventricular (nontraumatic) haemorrhage of fetus and newborn |
| P52.4Intracerebral (nontraumatic) haemorrhage of fetus and newborn |  | P52.4Intracerebral (nontraumatic) haemorrhage of fetus and newborn |
| P52.5Subarachnoid (nontraumatic) haemorrhage of fetus and newborn |  | P52.5Subarachnoid (nontraumatic) haemorrhage of fetus and newborn |
| P52.6Cerebellar (nontraumatic) and posterior fossa haemorrhage of fetus and newborn |  | P52.6Cerebellar (nontraumatic) and posterior fossa haemorrhage of fetus and newborn |
| P52.8Other intracranial (nontraumatic) haemorrhages of fetus and newborn |  | P52.8Other intracranial (nontraumatic) haemorrhages of fetus and newborn |
| P52.9Intracranial (nontraumatic) haemorrhage of fetus and newborn, unspecified |  | P52.9Intracranial (nontraumatic) haemorrhage of fetus and newborn, unspecified |
| P91.0 Neonatal cerebral ischemia |  | P91.0 Neonatal cerebral ischemia |
| P91.2 Neonatal cerebral leukomalacia |  | P91.2 Neonatal cerebral leukomalacia |
| P91.5 Neonatal coma |  | P91.5 Neonatal coma |
| P91.8 Other specified disturbances of cerebral status of newborn |  | P91.8 Other specified disturbances of cerebral status of newborn |
| P91.9 Disturbance of cerebral status of newborn, unspecified |  | P91.9 Disturbance of cerebral status of newborn, unspecified |

**Table S3: Read codes for case identification of non-traumatic acquired brain injury**

| **Read Codes** | | |
| --- | --- | --- |
| Eu13. ([X]Mental and behavioural dis due use sedatives/hypnotics) |  | F034H (Post hepatitis A vaccination encephalitis) |
| F0360 (Toxic encephalitis due to lead) |  | F034J (Post hepatitis B vaccination encephalitis) |
| F0361 (Toxic encephalitis due to mercury) |  | F034x (Post mixed vaccination encephalitis) |
| SP101 (Cerebral anoxia complication) |  | F034y (Post other specified vaccination encephalitis) |
| Q2… (Birth trauma, asphyxia and hypoxia) |  | F034z (Postimmunisation encephalitis NOS) |
| Q20.. (Birth trauma) |  | F035. (Postinfectious encephalitis) |
| Q489. (Acquired periventricular cysts of newborn) |  | F0350 (Encephalitis following chickenpox) |
| Q48A. (Neonatal cerebral leukomalacia) |  | F0351 (Encephalitis following measles) |
| Q208. (Cerebral oedema due to birth injury) |  | F035z (Postinfectious encephalitis NOS) |
| Q21.. (Intrauterine hypoxia and birth asphyxia) |  | F036. (Toxic encephalitis) |
| Q2121 (Liveborn with prelabour hypoxia) |  | F0360 (Toxic encephalitis due to lead) |
| Q2131 (Liveborn with labour hypoxia) |  | F0361 (Toxic encephalitis due to mercury) |
| Q215. (Severe birth asphyxia - Apgar score less than 4 at 1 minute) |  | F0362 (Toxic encephalitis due to thallium) |
| Q4111 (Intraventricular (nontraumatic) haemorrhage grade 2 fetus newborn) |  | F036z (Toxic encephalitis NOS) |
| Q4113 (Intraventricular haemorrhage due to birth injury) |  | F03X. (Bacterial meningoencephalitis+meningomyelitis,NEC) |
| Q412. (Perinatal subarachnoid haemorrhage) |  | F03y. (Other causes of encephalitis) |
| Q488. (Neonatal cerebral ischaemia) |  | F03z. (Encephalitis NOS) |
| Q200. (Subdural and cerebral haemorrhage due to birth trauma) |  | F04.. (Intracranial and intraspinal abscesses) |
| Q2000 (Cerebral haemorrhage unspecified, due to birth trauma) |  | F040. (Intracranial abscess) |
| S2001 (Subdural haemorrhage unspecified, due to birth trauma) |  | F0400 (Cerebral intracranial abscess) |
| Q2002 (Local subdural haematoma due to birth trauma) |  | F0401 (Cerebellar intracranial abscess) |
| Q2004 (Brain injury due to birth trauma NOS) |  | F0402 (Otogenic intracranial abscess) |
| Q2005 (Cerebral haematoma in fetus or newborn) |  | F0403 (Epidural intracranial abscess) |
| G664 (Cerebellar stroke syndrome) |  | F0404 (Extradural intracranial abscess) |
| G66.. (Stroke and cerebrovascular accident unspecified) |  | F0405 (Subdural intracranial abscess) |
| G663. (Brain stem stroke syndrome) |  | F0406 (Tuberculous intracranial abscess) |
| G68X. ( Sequelae of stroke, not specified as haemorrhage or infarction) |  | F040z (Intracranial abscess NOS) |
| Gyu6C ([X]Sequelae of stroke, not specified as haemorrhage or infarction) |  | F04z. (Intracranial or intraspinal abscess NOS) |
| F281. (Anoxic brain damage) |  | F06.. (Late effects of intracranial abscess or pyogenic infection) |
| SP100 ( Anoxic brain damage complication) |  | F113. (Acquired communicating hydrocephalus) |
| SP101 ( Cerebral anoxia complication) |  | F113z (Communicating hydrocephalus - acquired NOS) |
| L09y3 ( Cerebral anoxia following abortive pregnancy) |  | F114. (Acquired obstructive hydrocephalus) |
| A130. (Tuberculous meningitis) |  | F115. (Hydrocephalus) |
| A1302 (Tuberculous leptomeningitis) |  | F11x2 (Cerebral degeneration due to cerebrovascular disease) |
| A1303 (Tuberculous meningoencephalitis) |  | F11x3 (Cerebral degeneration due to congenital hydrocephalus) |
| A130z (Tuberculous meningitis NOS) |  | F11x5 (Cerebral degeneration due to myxoedema) |
| A131. (Tuberculoma of meninges) |  | F11x5 (Cerebral degeneration due to myxoedema) |
| A132. (Tuberculoma of brain) |  | F11x8 (Cerebral degeneration due to multifocal leukoencephalopathy) |
| A133. (Tuberculous abscess of brain) |  | F11x8 (Cerebral degeneration due to multifocal leukoencephalopathy) |
| A136. (Tuberculous encephalitis or myelitis) |  | F1322 (Myoclonic encephalopathy) |
| A1360 (Tuberculous encephalitis) |  | F1322 (Myoclonic encephalopathy) |
| A136z (Tuberculous encephalitis or myelitis NOS) |  | F212. (Acute and subacute haemorrhagic leukoencephalitis [Hurst]) |
| A206. (Plague meningitis) |  | F28.. (Other conditions of brain) |
| A2703 (Listerial cerebral arteritis) |  | F28.. (Other conditions of brain) |
| A36.. (Meningococcal infection) |  | F280. (Cerebral cysts) |
| A360. (Meningococcal meningitis) |  | F281. (Anoxic brain damage) |
| A361. (Meningococcal encephalitis) |  | F281. (Anoxic brain damage) |
| A36y. (Other specified meningococcal infection) |  | F283. (Unspecified encephalopathy) |
| A36yz (Other specified meningococcal infection NOS) |  | F283. (Unspecified encephalopathy) |
| A36z. (Meningococcal infection NOS) |  | F284. (Compression of brain) |
| A412. (Subacute sclerosing panencephalitis) |  | F284. (Compression of brain) |
| A413. (Progressive multifocal leukoencephalopathy) |  | F284z (Compression of brain NOS) |
| A413. (Progressive multifocal leukoencephalopathy) |  | F284z (Compression of brain NOS) |
| A42.. (Meningitis due to enterovirus) |  | F285. (Cerebral oedema) |
| A420. (Coxsackie viral meningitis) |  | F285. (Cerebral oedema) |
| A421. (ECHO viral meningitis) |  | F28y. (Other conditions of brain OS) |
| A42y. (Other specified viral meningitis) |  | F28y. (Other conditions of brain OS) |
| A42z. (Viral meningitis NOS) |  | F28yz (Other conditions of brain NOS) |
| A4y0. (Enteroviral encephalitis) |  | F28yz (Other conditions of brain NOS) |
| A4z0. (Lymphocytic choriomeningitis) |  | F2924 (Chemical meningitis) |
| A4z1. (Adenoviral meningitis) |  | F29y3 (Toxic encephalopathy) |
| A4zy0 (Acute inclusion body encephalitis) |  | Fyu00 ([X]Other bacterial meningitis) |
| A4zy1 (Acute necrotising encephalitis) |  | Fyu01 ([X]Meningitis in viral diseases classified elsewhere) |
| A4zy2 (Epidemic encephalitis) |  | Fyu02 ([X]Meningitis in mycoses classified elsewhere) |
| A4zy3 (Encephalitis lethargica) |  | Fyu03 ([X]Meningitis/other specified infectious parasitic diseases CE) |
| A4zy4 (Von Economo's encephalitis) |  | Fyu04 ([X]Meningitis due to other specified causes) |
| A4zy5 (Adenoviral encephalitis) |  | Fyu05 ([X]Bacterial meningoencephalitis+meningomyelitis,NEC) |
| A520. (Post varicella encephalitis) |  | Fyu06 ([X]Other encephalitis, myelitis and encephalomyelitis) |
| A530. (Herpes zoster with meningitis) |  | Fyu07 ([X]Encephalitis,myelitis+encephalomyelitis/bactrl disease CE) |
| A5314 (Zoster encephalitis) |  | Fyu08 ([X]Encephalitis,myelitis+encephalomyelitis/viral disease CE) |
| A543. (Herpetic meningoencephalitis) |  | Fyu0A ([X]Encephalitis,myelitis+encephalomyelitis/other diseases CE) |
| A54x1 (Herpes simplex meningitis) |  | Fyu0B ([X]Intracranial intraspinal abscess+granuloma in diseases CE) |
| A550. (Post measles encephalitis) |  | FyuA1 ([X]Other hydrocephalus) |
| A553. (Measles complicated by meningitis) |  | FyuA3 ([X]Hydrocephalus/infectious parasitic diseases CE) |
| A62.. (Mosquito-borne viral encephalitis) |  | FyuA4 ([X]Hydrocephalus in neoplastic disease classified elsewhere) |
| A620. (Japanese encephalitis) |  | FyuA5 ([X]Hydrocephalus in other diseases classified elsewhere) |
| A621. (Western equine encephalitis) |  | G6... (Cerebrovascular disease) |
| A622. (Eastern equine encephalitis) |  | G60.. (Subarachnoid haemorrhage) |
| A623. (St. Louis encephalitis) |  | G601. (Subarachnoid haemorrhage from carotid siphon and bifurcation) |
| A624. (Australian encephalitis) |  | G602. (Subarachnoid haemorrhage from middle cerebral artery) |
| A625. (California viral encephalitis) |  | G602. (Subarachnoid haemorrhage from middle cerebral artery) |
| A62y. (Other specified mosquito-borne virus encephalitis) |  | G603. (Subarachnoid haemorrhage from anterior communicating artery) |
| A62z. (Mosquito-borne viral encephalitis NOS) |  | G604. (Subarachnoid haemorrhage from posterior communicating artery) |
| A63.. (Tick-borne viral encephalitis) |  | G605. (Subarachnoid haemorrhage from basilar artery) |
| A630. (Russian spring-summer (taiga) encephalitis) |  | G606. (Subarachnoid haemorrhage from vertebral artery) |
| A631. (Louping ill encephalitis) |  | G60z. (Subarachnoid haemorrhage NOS) |
| A632. (Central European encephalitis) |  | G61.. (Intracerebral haemorrhage) |
| A63y. (Other tick-borne viral encephalitis) |  | G617. (Intracerebral haemorrhage, intraventricular) |
| A63y0 (Langat encephalitis) |  | G618. (Intracerebral haemorrhage, multiple localized) |
| A63y1 (Powassan encephalitis) |  | G61X. (Intracerebral haemorrhage in hemisphere, unspecified) |
| A63yz (Other tick-borne viral encephalitis NOS) |  | G61X0 (Left sided intracerebral haemorrhage, unspecified) |
| A63z. (Tick-borne viral encephalitis NOS) |  | G61X1 (Right sided intracerebral haemorrhage, unspecified) |
| A64.. (Viral encephalitis from other arthropods) |  | G61z. (Intracerebral haemorrhage NOS) |
| A721. (Mumps meningitis) |  | G63.. (Precerebral arterial occlusion) |
| A722. (Mumps encephalitis) |  | G630. (Basilar artery occlusion) |
| A791. (ECHO virus) |  | G631. (Carotid artery occlusion) |
| A792. (Coxsackie virus) |  | G632. (Vertebral artery occlusion) |
| A9041 (Congenital syphilitic encephalitis) |  | G633. (Multiple and bilateral precerebral arterial occlusion) |
| A9042 (Congenital syphilitic meningitis) |  | G63y. (Other precerebral artery occlusion) |
| A9180 (Acute secondary syphilitic meningitis) |  | G63y0 (Cerebral infarct due to thrombosis of precerebral arteries) |
| A942. (Syphilitic meningitis) |  | G63y1 (Cerebral infarction due to embolism of precerebral arteries) |
| A94y0 (Syphilitic encephalitis) |  | G63z. (Precerebral artery occlusion NOS) |
| A94y6 (Rupture of syphilitic cerebral aneurysm) |  | G64.. (Cerebral arterial occlusion) |
| A98y1 (Gonococcal meningitis) |  | G640. (Cerebral thrombosis) |
| AA0y0 (Leptospiral meningitis) |  | G6400 (Cerebral infarction due to thrombosis of cerebral arteries) |
| AB2y2 (Candidal meningitis) |  | G641. (Cerebral embolism) |
| AB32. (Coccidioidal meningitis) |  | G6410 (Cerebral infarction due to embolism of cerebral arteries) |
| AB401 (Histoplasma capsulatum with meningitis) |  | G64z. (Cerebral infarction NOS) |
| AB411 (Histoplasma duboisii with meningitis) |  | G64z2 (Left sided cerebral infarction) |
| AB4z1 (Histoplasmosis with meningitis) |  | G64z3 (Right sided cerebral infarction) |
| AB652 (Cryptococcal meningitis) |  | G67.. (Other cerebrovascular disease) |
| AD00. (Toxoplasma meningoencephalitis) |  | G670. (Cerebral atherosclerosis) |
| AE20. (Late effects of viral encephalitis) |  | G671. (Generalised ischaemic cerebrovascular disease NOS) |
| Ayu3C ([X]Other meningococcal infections) |  | G672. (Hypertensive encephalopathy) |
| Ayu3D ([X]Meningococcal infection, unspecified) |  | G672. (Hypertensive encephalopathy) |
| Ayu85 ([X]Other mosquito-borne viral encephalitis) |  | G674. (Cerebral arteritis) |
| Ayu86 ([X]Mosquito-borne viral encephalitis, unspecified) |  | G675. (Moya Moya disease) |
| Ayu87 ([X]Other tick-borne viral encephalitis) |  | G676. (Nonpyogenic venous sinus thrombosis) |
| Ayu88 ([X]Tick-borne viral encephalitis, unspecified) |  | G6760 (Cerebral infarction due to cerebral venous thrombosis, nonpyogenic) |
| Ayu89 ([X]Arthropod-borne viral encephalitis, unspecified) |  | G6770 (Occlusion and stenosis of middle cerebral artery) |
| Ayu8A ([X]Other specified viral encephalitis) |  | G6771 (Occlusion and stenosis of anterior cerebral artery) |
| Ayu8B ([X]Unspecified viral encephalitis) |  | G6772 (Occlusion and stenosis of posterior cerebral artery) |
| Ayu8C ([X]Other viral meningitis) |  | G6774 (Occlusion stenosis of multiple and bilat cerebral arteries) |
| Ayu8D ([X]Viral meningitis, unspecified) |  | G67y. (Other cerebrovascular disease OS) |
| AyuJ8 ([X]Sequelae of viral encephalitis) |  | G67z. (Other cerebrovascular disease NOS) |
| B51.. (Malignant neoplasm of brain) |  | G68.. (Late effects of cerebrovascular disease) |
| B5101 (Malignant neoplasm of cerebral cortex) |  | G680. (Sequelae of subarachnoid haemorrhage) |
| B510z (Malignant neoplasm of cerebrum NOS) |  | G681. (Sequelae of intracerebral haemorrhage) |
| B511. (Malignant neoplasm of frontal lobe) |  | G683. (Sequelae of cerebral infarction) |
| B512. (Malignant neoplasm of temporal lobe) |  | G68W. (Sequelae/other + unspecified cerebrovascular diseases) |
| B512z (Malignant neoplasm of temporal lobe NOS) |  | G68X. (Sequelae of stroke, not specified as haemorrhage or infarction) |
| B513. (Malignant neoplasm of parietal lobe) |  | G6y.. (Other specified cerebrovascular disease) |
| B515. (Malignant neoplasm of cerebral ventricles) |  | G6z.. (Cerebrovascular disease NOS) |
| B5151 (Malignant neoplasm of floor of cerebral ventricle) |  | Gyu6. ([X]Cerebrovascular diseases) |
| B515z (Malignant neoplasm of cerebral ventricle NOS) |  | Gyu60 ([X]Subarachnoid haemorrhage from other intracranial arteries) |
| B516. (Malignant neoplasm of cerebellum) |  | Gyu61 ([X]Other subarachnoid haemorrhage) |
| B517. (Malignant neoplasm of brain stem) |  | Gyu62 ([X]Other intracerebral haemorrhage) |
| B5170 (Malignant neoplasm of cerebral peduncle) |  | Gyu64 ([X]Other cerebral infarction) |
| B5172 (Malignant neoplasm of midbrain) |  | Gyu65 ([X]Occlusion and stenosis of other precerebral arteries) |
| B517z (Malignant neoplasm of brain stem NOS) |  | Gyu66 ([X]Occlusion and stenosis of other cerebral arteries) |
| B51y. (Malignant neoplasm of other parts of brain) |  | Gyu67 ([X]Other specified cerebrovascular diseases) |
| B51yz (Malignant neoplasm of other part of brain NOS) |  | Gyu68 ([X]Cerebral arteritis in infectious and parasitic diseases) |
| B51z. (Malignant neoplasm of brain NOS) |  | Gyu69 ([X]Cerebral arteritis in other diseases CE) |
| B520. (Malignant neoplasm of cranial nerves) |  | Gyu6A ([X]Other cerebrovascular disorders in diseases CE) |
| B520z (Malignant neoplasm of cranial nerves NOS) |  | Gyu6D ([X]Sequelae/other + unspecified cerebrovascular diseases) |
| B521. (Malignant neoplasm of cerebral meninges) |  | Gyu6F ([X]Intracerebral haemorrhage in hemisphere, unspecified) |
| B5210 (Malignant neoplasm of cerebral dura mater) |  | H27y0 (Influenza with encephalopathy) |
| B5211 (Malignant neoplasm of cerebral arachnoid mater) |  | H27y0 (Influenza with encephalopathy) |
| B5212 (Malignant neoplasm of cerebral pia mater) |  | L2637 (Maternal care for fetal hypoxia) |
| B521z (Malignant neoplasm of cerebral meninges NOS) |  | L417. (Obstetric cerebral venous thrombosis) |
| B52X. (Malignant neoplasm of meninges, unspecified) |  | L4170 (Cerebral venous thrombosis in pregnancy) |
| B583. (Secondary malignant neoplasm of brain and spinal cord) |  | L4171 (Cerebral venous thrombosis in the puerperium) |
| B5830 (Secondary malignant neoplasm of brain) |  | P10.. (Spina bifida with hydrocephalus) |
| B583z (Secondary malignant neoplasm of brain or spinal cord NOS) |  | P100. (Unspecified spina bifida with hydrocephalus) |
| B7F.. (Benign neoplasm of brain and other parts of nervous system) |  | P1000 (Spina bifida with hydrocephalus, unspecified) |
| B7F0. (Benign neoplasm of brain) |  | P1001 (Cervical spina bifida with hydrocephalus) |
| B7F00 (Benign neoplasm of brain, supratentorial) |  | P1002 (Thoracic spina bifida with hydrocephalus) |
| B7F1. (Benign neoplasm of cranial nerves) |  | P1003 (Lumbar spina bifida with hydrocephalus) |
| B7F2. (Benign neoplasm of cerebral meninges) |  | P100z (Spina bifida with hydrocephalus NOS) |
| B7F2z (Benign neoplasm of cerebral meninges NOS) |  | P102. (Spina bifida with hydrocephalus - open) |
| B7FX. (Benign neoplasm of meninges, unspecified) |  | P1020 (Unspecified spina bifida with hydrocephalus - open) |
| B7Fz. (Benign neoplasm of brain or other nervous system NOS) |  | P1021 (Cervical spina bifida with hydrocephalus - open) |
| B925. (Neoplasm of uncertain behaviour of brain and spinal cord) |  | P1022 (Thoracic spina bifida with hydrocephalus - open) |
| B9250 (Neoplasm of uncertain behaviour of brain) |  | P1023 (Lumbar spina bifida with hydrocephalus - open) |
| B9252 (Neoplasm of uncertain or unknown behaviour brain, supratentorial) |  | P1024 (Sacral spina bifida with hydrocephalus - open) |
| B925z (Neoplasm of uncertain behaviour of brain or spinal cord NOS) |  | P102z (Spina bifida with hydrocephalus - open NOS) |
| B926. (Neoplasm of uncertain behaviour of meninges) |  | P103. (Spina bifida with hydrocephalus - closed) |
| B9260 (Neoplasm of uncertain behaviour of cerebral meninges) |  | P1030 (Unspecified spina bifida with hydrocephalus - closed) |
| B926z (Neoplasm of uncertain behaviour of meninges NOS) |  | P1031 (Cervical spina bifida with hydrocephalus - closed) |
| B92z0 (Neoplasm of uncertain behaviour of cranial nerves) |  | P1032 (Thoracic spina bifida with hydrocephalus - closed) |
| BA06. (Neoplasm of unspecified nature of brain) |  | P1033 (Lumbar spina bifida with hydrocephalus - closed) |
| BBd.. ([M]Meningiomas) |  | P1034 (Sacral spina bifida with hydrocephalus - closed) |
| BBd.. ([M]Meningiomas) |  | P103z (Spina bifida with hydrocephalus - closed NOS) |
| BBd0. ([M]Meningioma NOS) |  | P104. (Spina bifida with hydrocephalus of late onset) |
| BBd0. ([M]Meningioma NOS) |  | P10y. (Other specified spina bifida with hydrocephalus) |
| BBd1. ([M]Meningiomatosis NOS) |  | P10yz (Other spina bifida with hydrocephalus NOS) |
| BBd1. ([M]Meningiomatosis NOS) |  | P2401 (Multiple congenital cerebral cysts) |
| BBd2. ([M]Meningioma, malignant) |  | P7y01 (Congenital cerebral arteriovenous aneurysm) |
| BBd2. ([M]Meningioma, malignant) |  | Pyu04 ([X]Unspecified spina bifida with hydrocephalus) |
| BBd3. ([M]Meningotheliomatous meningioma) |  | Q2... (Birth trauma, asphyxia and hypoxia) |
| BBd3. ([M]Meningotheliomatous meningioma) |  | Q200. (Subdural and cerebral haemorrhage due to birth trauma) |
| BBd4. ([M]Fibrous meningioma) |  | Q2000 (Cerebral haemorrhage unspecified, due to birth trauma) |
| BBd4. ([M]Fibrous meningioma) |  | Q2007 (Cerebral haemorrhage due to birth injury) |
| BBd5. ([M]Psammomatous meningioma) |  | Q200y (Subdural or cerebral haemorrhage due to birth trauma OS) |
| BBd5. ([M]Psammomatous meningioma) |  | Q200z (Subdural or cerebral haemorrhage due to birth trauma NOS) |
| BBd6. ([M]Angiomatous meningioma) |  | Q208. (Cerebral oedema due to birth injury) |
| BBd6. ([M]Angiomatous meningioma) |  | Q208. (Cerebral oedema due to birth injury) |
| BBd7. ([M]Haemangioblastic meningioma) |  | Q21.. (Intrauterine hypoxia and birth asphyxia) |
| BBd7. ([M]Haemangioblastic meningioma) |  | Q2121 (Liveborn with prelabour hypoxia) |
| BBd8. ([M]Haemangiopericytic meningioma) |  | Q2131 (Liveborn with labour hypoxia) |
| BBd8. ([M]Haemangiopericytic meningioma) |  | Q2141 (Liveborn with fetal hypoxia, unspecified) |
| BBd9. ([M]Transitional meningioma) |  | Q215. (Severe birth asphyxia - Apgar score less than 4 at 1 minute) |
| BBd9. ([M]Transitional meningioma) |  | Q216. (Mild to moderate birth asphyxia - Apgar score 4-7 at 1 min) |
| BBdA. ([M]Papillary meningioma) |  | Q21z. (Liveborn with birth asphyxia NOS) |
| BBdA. ([M]Papillary meningioma) |  | Q2y.. (Other specified birth trauma, asphyxia or hypoxia) |
| ByuA. ([X]Malignant neoplasm of eye, brain and other parts of cent) |  | Q2z.. (Birth trauma, asphyxia or hypoxia NOS) |
| ByuA2 ([X]Malignant neoplasm of meninges, unspecified) |  | Q412. (Perinatal subarachnoid haemorrhage) |
| ByuGP ([X]Benign neoplasm of meninges, unspecified) |  | Q4120 (Subarachnoid haemorrhage due to birth injury) |
| C102. (Diabetes mellitus with hyperosmolar coma) |  | Q4170 (Intracerebral (nontraumatic) haemorrhage of fetus and newborn) |
| C1020 (Diabetes mellitus, juvenile type, with hyperosmolar coma) |  | Q4370 (Bilirubin encephalopathy) |
| C1021 (Diabetes mellitus, adult onset, with hyperosmolar coma) |  | Q4370 (Bilirubin encephalopathy) |
| C102z (Diabetes mellitus NOS with hyperosmolar coma) |  | S62.. (Cerebral haemorrhage following injury) |
| C103. (Diabetes mellitus with ketoacidotic coma) |  | S62z. (Cerebral haemorrhage following injury NOS) |
| C1030 (Diabetes mellitus, juvenile type, with ketoacidotic coma) |  | S63.. (Other cerebral haemorrhage following injury) |
| C1031 (Diabetes mellitus, adult onset, with ketoacidotic coma) |  | S63z. (Other cerebral haemorrhage following injury NOS) |
| C103y (Other specified diabetes mellitus with coma) |  | SN41. (Drowning and nonfatal submersion) |
| C103z (Diabetes mellitus NOS with ketoacidotic coma) |  | SN413 (Drowning) |
| C10A0 (Malnutrition-related diabetes mellitus with coma) |  | SN41z (Drowning or nonfatal submersion NOS) |
| C110. (Hypoglycaemic coma) |  | SN47. ( Asphyxiation and strangulation) |
| C110z (Hypoglycaemic coma NOS) |  | SN47. (Asphyxiation and strangulation) |
| Eu061 ([X]Postencephalitic syndrome) |  | SN470 ( Suffocation, unspecified) |
| F00.. (Bacterial meningitis) |  | SN470 (Suffocation, unspecified) |
| F000. (Haemophilus meningitis) |  | SN471 (Suffocation by strangulation) |
| F001. (Pneumococcal meningitis) |  | SN472 (Suffocation by bedclothes) |
| F002. (Streptococcal meningitis) |  | SN473 (Suffocation by cave-in) |
| F003. (Staphylococcal meningitis) |  | SN474 (Suffocation by mechanical cause) |
| F004. (Meningitis - tuberculous) |  | SN475 (Suffocation by plastic bag) |
| F005. (Meningitis - meningococcal) |  | SN476 (Suffocation by pressure) |
| F007. (Meningitis in other bacterial disease classified elsewhere) |  | SN47z (Asphyxiation or strangulation NOS) |
| F0070 (Meningitis due to gonococcus) |  | SN47z (Asphyxiation or strangulation NOS) |
| F0071 (Meningitis due to listeriosis) |  | TF... (Accidents caused by submersion, suffocation and foreign bodies) |
| F0072 (Meningitis due to neurosyphilis) |  | TF0.. (Accidental drowning and submersion) |
| F0073 (Meningitis due to salmonella) |  | TF00. (Accidental drowning and submersion while water skiing) |
| F0074 (Meningitis due to congenital syphilis) |  | TF01. (Accidental drowning and submersion during diving sport) |
| F0075 (Meningitis due to secondary syphilis) |  | TF010 (Accidental drowning and submersion while scuba diving NOS) |
| F0076 (Meningitis due to typhoid fever) |  | TF011 (Accidental drowning and submersion while skin diving NOS) |
| F0077 (Meningitis due to actinomycosis) |  | TF012 (Accidental drowning/submersion-underwater spear fishing NOS) |
| F0078 (Meningitis due to pertussis) |  | TF01z (Accidental drowning and submersion during diving sport NOS) |
| F007z (Unspecified meningitis in bacterial disease EC) |  | TF02. (Accidental drowning and submersion while engaged in sport or recreational activity without diving equipment) |
| F00y. (Other specified bacterial meningitis) |  | TF020 (Accidental drowning/submersion-fishing not from boat/diving) |
| F00y0 (Meningitis due to Aerobacter aerogenes) |  | TF021 (Accidental drowning/submersion-hunting not from boat/diving) |
| F00y1 (Meningitis due to bacillus pyocyaneus) |  | TF022 (Accidental drowning and submersion while ice skating) |
| F00y2 (Meningitis due to Escherichia coli) |  | TF023 (Accidental drowning and submersion while playing in water) |
| F00y3 (Meningitis due to Friedlander bacillus) |  | TF024 (Accidental drowning and submersion while surfboarding) |
| F00y4 (Meningitis due to klebsiella pneumoniae) |  | TF025 (Accidental drowning and submersion while wading in water) |
| F00y5 (Meningitis due to proteus morganii) |  | TF026 (Accidental drowning and submersion while swimming NOS) |
| F00y6 (Meningitis due to pseudomonas) |  | TF02z (Accidental drowning and submersion during sport NOS) |
| F00yz (Other specified bacterial meningitis NOS) |  | TF03. (Accidental drowning/submersion-swim/diving-not sport/recreation) |
| F00z. (Bacterial meningitis NOS) |  | TF030 (Accidental drowning and submersion during marine salvage) |
| F01.. (Meningitis due to other organisms) |  | TF031 (Accidental drowning and submersion while pearl diving) |
| F010. (Meningitis due to fungal organisms) |  | TF032 (Accidental drowning/submersion during placement fishing nets) |
| F0100 (Meningitis due to cryptococcus) |  | TF033 (Accidental drowning/submersion during rescue attempt person) |
| F010z (Other fungal meningitis) |  | TF035 (Accidental drowning/submersion during underwater repairs) |
| F011. (Meningitis due to viral organisms EC) |  | TF03z (Accidental drowning/submersion-swim/diving, not sport NOS) |
| F0110 (Meningitis due to coxsackie virus) |  | TF04. (Accidental drowning and submersion in bathtub) |
| F0111 (Meningitis due to ECHO virus) |  | TF0y. (Other accidental drowning and submersion) |
| F0112 (Meningitis due to herpes zoster virus) |  | TF0y0 (Accidental drowning and submersion in quenching tank) |
| F0113 (Meningitis due to herpes simplex virus) |  | TF0y1 (Accidental drowning and submersion in swimming pool) |
| F0114 (Meningitis due to mumps virus) |  | TF0yz (Other accidental drowning and submersion NOS) |
| F0115 (Meningitis due to lymphocytic choriomeningitis virus) |  | TF0z. (Accidental drowning and submersion NOS) |
| F0116 (Meningitis due to adenovirus) |  | TF1.. (Inhalation and ingestion of food causing obstruction of respiratory tract or suffocation) |
| F0117 (Varicella meningitis) |  | TF10. (Asphyxia by food) |
| F011y (Other viral meningitis) |  | TF100 (Asphyxia by bone in food) |
| F011z (Meningitis - viral NOS) |  | TF101 (Asphyxia by seed in food) |
| F012. (Meningitis due to trypanosomiasis) |  | TF102 (Asphyxia by regurgitated food) |
| F013. (Meningitis due to sarcoidosis) |  | TF10z (Asphyxia by food NOS) |
| F01y. (Other non-bacterial meningitis) |  | TF12. (Suffocation by food) |
| F01y0 (Meningitis due to Leptospira) |  | TF120 (Suffocation by bone in food) |
| F01yz (Other non-bacterial meningitis NOS) |  | TF121 (Suffocation by seed in food) |
| F01z. (Meningitis due to organism NOS) |  | TF122 (Suffocation by regurgitated food) |
| F02.. (Meningitis of unspecified cause) |  | TF12z (Suffocation by food NOS) |
| F020. (Nonpyogenic meningitis) |  | TF1z. (Inhalation/ingestion food - resp obstruction/suffocation NOS) |
| F021. (Eosinophilic meningitis) |  | TF2.. (Inhalation and ingestion of other object causing obstruction of respiratory tract or suffocation) |
| F022. (Chronic meningitis) |  | TF2z. (Inhalation/ingestion obj NOS + resp obstruction/suffocation) |
| F024. (Benign recurrent meningitis) |  | TF3.. (Accidental mechanical suffocation) |
| F02z. (Unspecified meningitis) |  | TF30. (Accidental mechanical suffocation in bed or cradle) |
| F03.. (Encephalitis, myelitis and encephalomyelitis) |  | TF300 (Accidental mechanical suffocation in bed) |
| F030. (Encephalitis in viral disease EC) |  | TF301 (Accidental mechanical suffocation in cradle) |
| F0300 (Encephalitis due to kuru) |  | TF30z (Accidental mechanical suffocation in bed NOS) |
| F0301 (Encephalitis due to subacute sclerosing panencephalitis) |  | TF31. (Accidental mechanical suffocation by plastic bag) |
| F0302 (Encephalitis due to poliomyelitis) |  | TF32. (Accidental mechanical suffocation - lack of air/closed space) |
| F0303 (Encephalitis due to arthropod-borne virus) |  | TF320 (Accidental mechanical suffocation in refrigerator) |
| F0304 (Encephalitis due to herpes simplex virus) |  | TF321 (Accidental mechanical suffocation diving, insufficient air) |
| F0305 (Encephalitis due to mumps virus) |  | TF32z (Accidental mechanical suffocation, lack air/closed space NOS) |
| F0306 (Encephalitis due to rubella virus) |  | TF33. (Accidental mechanical suffocation by falling earth etc) |
| F030z (Encephalitis in viral disease NOS) |  | TF33z (Accidental mechanical suffocation by falling earth etc NOS) |
| F031. (Encephalitis due to rickettsia EC) |  | TF3y. (Other accidental mechanical suffocation) |
| F032. (Encephalitis due to protozoa EC) |  | TF3yz (Other accidental mechanical suffocation NOS) |
| F0320 (Encephalitis due to malaria) |  | TF3z. (Accidental mechanical suffocation, unspecified) |
| F0321 (Encephalitis due to trypanosomiasis) |  | TF3z0 (Accidental mechanical asphyxia NOS) |
| F032z (Encephalitis due to protozoa EC NOS) |  | TF3z1 (Accidental strangulation NOS) |
| F033. (Encephalitis due to other infection EC) |  | TF3z2 (Accidental suffocation NOS) |
| F0330 (Encephalitis due to meningococcus) |  | TF3zz (Accidental mechanical suffocation NOS) |
| F0331 (Encephalitis due to congenital syphilis) |  | TFz.. (Accidents caused by submersion, suffocation ,foreign body NOS) |
| F0332 (Encephalitis due to syphilis unspecified) |  | TG30A (Accidental mechanical suffocation caused by machinery) |
| F0333 (Encephalitis due to tuberculosis) |  | TK3.. (Suicide and self inflicted injury by hanging, strangulation and suffocation) |
| F0334 (Encephalitis due to toxoplasmosis) |  | TK3.. (Suicide and self inflicted injury by hanging, strangulation and suffocation) |
| F033z (Unspecified encephalitis due to other infection EC) |  | TK31. (Suicide + self inflicted injury by suffocation by plastic bag) |
| F034. (Postimmunisation encephalitis) |  | TK4.. (Suicide and self inflicted injury by drowning) |
| F0340 (Post BCG vaccination encephalitis) |  | TL3 (Assault by hanging and strangulation) |
| F0341 (Post typhoid vaccination encephalitis) |  | TL3z. (Assault by hanging or strangulation NOS) |
| F0342 (Post paratyphoid vaccination encephalitis) |  | TL32. (Assault by strangulation) |
| F0343 (Post cholera vaccination encephalitis) |  | TL33. (Assault by suffocation) |
| F0344 (Post plague vaccination encephalitis) |  | TL4.. (Assault by drowning) |
| F0345 (Post tetanus vaccination encephalitis) |  | TN3.. ( Injury undetermined whether accidentally or purposely inflicted, hanging, strangulation and suffocation) |
| F0346 (Post diphtheria vaccination encephalitis) |  | TN3.. (Injury undetermined whether accidentally or purposely inflicted, hanging, strangulation and suffocation) |
| F0347 (Post pertussis vaccination encephalitis) |  | TN3.. (Injury ?accidental, hanging, strangulation and suffocation) |
| F0348 (Post smallpox vaccination encephalitis) |  | TN31. (Injury ?accidental, suffocation by plastic bag) |
| F0349 (Post rabies vaccination encephalitis) |  | TN3z. (Injury ?accidental, hanging/strangulation/suffocation NOS) |
| F034A (Post typhus vaccination encephalitis) |  | TN4.. (Injury undetermined whether accidentally or purposely inflicted, drowning) |
| F034B (Post yellow fever vaccination encephalitis) |  | SF00. (Crush injury, face) |
| F034C (Post measles vaccination encephalitis) |  | SF03. (Crushing injury of skull) |
| F034D (Post polio vaccination encephalitis) |  | SF0X. (Crushing injury of head, part unspecified) |
| F034E (Post mumps vaccination encephalitis) |  | Syu0F ([X]Crushing injury of other parts of head) |
| F034F (Post rubella vaccination encephalitis) |  | Syu0G ([X]Crushing injury of head, part unspecified) |
| F034G (Post influenza vaccination encephalitis) |  |  |

**Table S4: Socrates dataset codes for non-traumatic acquired brain injury**

| **Socrates Codes** |
| --- |
| 1803 (brain injury cause not specified) |
| 2401 (stroke, brain / subarachnoid haemorrhage, cerebrovascular accident (CVA), parietal / cerebral / cerebellar / middle cerebral artery (MCA) / thalamic / frontal lobe infarct) |
| 1002 (birth asphyxia or intrauterine hypoxia) |
